# Healthcare Resource Utilization (HCRU) and Direct Medical Costs Associated with Long COVID or Post-COVID-19 Conditions: Findings from a Literature Review

**DOI:** 10.3390/jmahp13010007

**Published:** 2025-02-12

**Authors:** Elżbieta Łukomska, Krzysztof Kloc, Malwina Kowalska, Aleksandra Matjaszek, Keya Joshi, Stefan Scholz, Nicolas Van de Velde, Ekkehard Beck

**Affiliations:** 1 Clever-Access, 30-415 Krakow, Poland; 2 Moderna, Inc., Cambridge, MA 02142, USA; 3 Moderna, 80333 Munich, Germany

**Keywords:** SARS-CoV-2, COVID-19, long COVID, post-COVID-19, healthcare costs, healthcare resource use, inpatient admission, hospitalization, outpatient visit, emergency room

## Abstract

Approximately 10–20% of individuals suffering from COVID-19 develop prolonged symptoms known as long COVID or post-COVID condition (LC). This review aimed to assess healthcare resource use (HCRU) and healthcare costs associated with LC. Because LC is not clearly defined and often remains undiagnosed, studies reporting on long-term follow-up of individuals with a COVID-19 diagnosis were also included. Among the 41 publications included, 36 reported on HCRU and 16 on costs. Individuals with LC had significantly elevated HCRU and healthcare costs vs. controls without a COVID-19 diagnosis over ≥15 months, with a 7.6–13.1% increase in total healthcare costs per person per month as assessed by difference-in-difference analysis. Among studies that did not specifically refer to LC, having a COVID-19 diagnosis was associated with a significant 4–10% increase in long-term total HCRU over 6–8 months and a 1.3- to 2.9-fold relative increase in total healthcare costs over 6 months. Due to the heterogeneity of the included studies, high-quality evidence is needed to better understand the economic burden of LC. In the absence of effective treatments, prioritizing the prevention of acute COVID-19, e.g., through vaccination, may be crucial for preventing LC and the associated long-term HCRU and medical spending.

## 1. Introduction

As of 21 April 2024, the World Health Organization (WHO) reported 775.4 million confirmed COVID-19 cases and more than 7 million confirmed deaths from the disease [1], which has been estimated to cost the global economy US $13.8 trillion [2]. In December 2020, the US Food and Drug Administration (FDA) and the European Medicines Agency (EMA) issued emergency use/conditional authorizations for the first vaccines against COVID-19 [3] and, during the following three years, 13.6 billion doses of vaccines against COVID-19 were administered globally [1].

Many individuals with COVID-19 develop mild to moderate illness and recover without hospitalization [4]. Common symptoms include fever, cough, tiredness, and loss of taste and/or smell [4]. Although most people who develop COVID-19 recover fully, approximately 10–20% develop prolonged symptoms known as long COVID or post-COVID-19 conditions [5]. However, the prevalence of prolonged COVID-19 symptoms has been reported to be as high as 45% [6]. Common symptoms in individuals with long COVID include fatigue, cognitive dysfunction, shortness of breath, sleep disorder, persistent cough, chest pain, trouble speaking, loss of smell or taste, myalgia, and fever [5,7]. However, the number and severity of symptoms vary between affected individuals [8], and many of the symptoms are non-specific, resulting in difficulty in diagnosing long COVID [9] and uncertainty around its true prevalence [10]. Nonetheless, long COVID may impact the everyday lives of affected individuals, who report impaired quality of life [11,12,13,14] as well as adverse effects of the condition on employment [11,13,15,16], disability status [9], social and family life [11,13], and mental health [11,13]. In the US alone, annual wages lost due to long COVID approach US $200 billion [17], while the total cost of long COVID to the US economy has been estimated at US $3.7 trillion, assuming each individual case of long COVID lasts 5 years [18]. In the European Union, long COVID was estimated to reduce labor supply by 364,000–663,000 in 2021 and 621,000–1,112,000 person-equivalents in 2022 due to the combined effects of lower productivity, increased sick leaves, reduced hours, and increased unemployment [19].

Despite the substantial burden associated with long COVID, policy decision-makers still face gaps in evidence around long COVID and its impact [20] although the understanding of the condition is rapidly evolving [9]. Whereas many studies aim to address this uncertainty by improving the understanding of the disease burden associated with long COVID from a health outcomes and macroeconomic perspective, less is known about healthcare resource use (HCRU) and direct medical cost impacts, which are crucial to help inform governments and payers in their efforts to adequately provide care to patients with long COVID and holistically mitigate the current and future burden associated with long COVID. Consequently, we conducted a comprehensive review of the available peer-reviewed and gray literature to assess long-term costs and HCRU following SARS-CoV-2 infection, as well as to help understand the methodological approaches used in current studies, which can provide helpful insights for future evidence-generation efforts.

## 2. Materials and Methods

We conducted a comprehensive literature review that used a three-pronged approach combining searches of (1) the PubMed database, (2) Health Technology Assessment (HTA) Agency websites, and (3) additional data sources.

The PubMed search utilized a comprehensive set of condition- and outcome-specific keywords and was conducted on 21 November 2023. Please see Supplementary Table S1 for the full search strategy.

The websites of HTA agencies operating in diverse jurisdictions and international organizations were searched in November 2023. See Supplementary Table S2 for a full list of the organizations included in this search.

The additional data sources included hand searches of the Google Scholar database (searched in December 2023) and the preprint servers medRxiv and Social Science Research Network (SSRN) (searched in November 2023). Please see Supplementary Table S3 for details of the keywords used to search Google Scholar and the preprint servers. In addition, the reference lists of included publications were reviewed to identify any relevant studies that were not already included.

The eligibility criteria for the review are summarized in Table 1. The review focused on observational studies reporting on long-term healthcare costs and/or HCRU incurred after SARS-CoV-2 infection. The broad population eligibility criteria used in this review should be noted. Several definitions of long COVID or post-COVID-19 conditions exist. The US Department of Health and Human Services (HHS) and the Centers for Disease Control and Prevention (CDC) define long COVID broadly as signs, symptoms, and conditions that continue or develop after initial SARS-CoV-2 infection and are present at ≥4 weeks after the initial infection phase [21]. In contrast, the WHO defines long COVID as the continuation or development of new symptoms 3 months after the initial SARS-CoV-2 infection, with these symptoms lasting for at least 2 months with no other explanation [22]. Considering the lack of a uniform definition of long COVID, the eligibility criteria for the review were very broad to ensure that the relevant evidence was comprehensively captured. Studies using any definition of long COVID or post-COVID-19 condition were included, and the definitions were extracted, as were studies that did not explicitly refer to long COVID but provided follow-up data for patients after the acute phase of SARS-CoV-2 infection (Table 1). Titles and abstracts of studies captured in the search were screened by a single reviewer. Potentially eligible records progressed to full-text review which was also conducted by a single reviewer. Data from studies meeting the eligibility criteria were extracted by two analysts into a Microsoft Excel (version 2412) file. Information was collected on methodology, design, population, assessed arm(s), and main results of the studies. This report follows the reporting principles set out in the Preferred Reporting Items for Systematic Reviews and Meta-Analyses (PRISMA) statement [16].

## 3. Results

### 3.1. Overview of Included Studies

**Figure 1:**
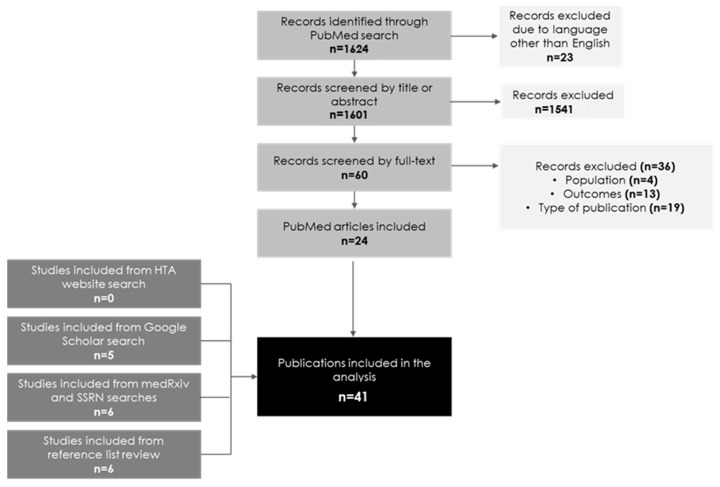
PRISMA flow diagram. Abbreviations: SSRN, Social Science Research Network.

A total of 41 publications were included in the review. Of those, 24 articles were identified through the PubMed search and 17 through searching additional data sources (Figure 1). No relevant publications were identified through searching the websites of HTA agencies.

Most of the analyzed studies were retrospective (93%), while only 3 studies were prospective (7%).

Fifty-six percent of the studies (n = 23) evaluated the adult population, 15 studies (37%) reported on a mixed population, and 3 studies (7%) focused solely on children. The designs of the included studies were highly heterogeneous. Twenty-nine studies had at least one control group and 12 studies were single-arm. The most common control group was the group without a COVID-19 diagnosis used in 18 studies, followed by “no long COVID” (although previously diagnosed with COVID-19) in 6 studies, and “not hospitalized for COVID-19” in 3 studies. The remaining control groups featured in single studies; examples include “pre-pandemic” and “long flu”.

In terms of geographic location, cost or HCRU data were identified for 14 different countries in total; most studies were conducted in the US (15 studies), followed by the UK (7 studies) and Italy (3 studies).

Information on SARS-CoV-2 strains was available in only seven studies (17%) reporting on 1–3 strains per study, mostly pre-Omicron (4 studies) and Delta strains (3 studies). One study reported solely on long COVID due to the Omicron strain [23].

Follow-up was highly variable. Six studies reported median follow-up, which ranged from 63 days to 26 months. Mean follow-up was reported in five studies and ranged from 140 days to 395 days. Eleven studies had a pre-defined duration that ranged from 2 to 12 months. The most common observation periods were 6 months (5 studies) and 12 months (4 studies). The minimum follow-up duration was provided in three studies and was approximately 90 days. The maximum follow-up duration was described in 11 studies and ranged from 90 days to 2 years. Follow-up of studies with the definition of long COVID most often started 3 months (5 studies) or 1 month after COVID-19 diagnosis (4 studies) or it was not clearly stated (in seven studies with diagnosis based on coding systems). Follow-up in studies that reported on HCRU/costs after the acute COVID-19 phase but did not refer explicitly to long COVID usually started 1 month (9 studies), 2–4 months (3 studies), 2 weeks (2 studies) after COVID-19 diagnosis, or 0–6 weeks after discharge from COVID-related hospitalization (4 studies).

### 3.2. Definitions of Long COVID Used in Included Studies

As noted above, the review included both studies that considered long COVID specifically and those that did not refer to long COVID but provided long-term follow-up data post-acute COVID-19 phase. Long COVID was considered in 19 studies (46%), with the definition based on symptoms in 12 studies (57%), on coding in 8 studies (38%), and on another type of definition in 1 study (5%). Two studies provided both symptom-based and coding-based definitions [24,25]. The specific definitions of long COVID used in the included studies were highly heterogeneous and are listed in Supplementary Table S4.

Among studies that used a symptom-based definition, most long COVID definitions required the presence of symptoms for at least 4 weeks after COVID-19 diagnosis, consistent with the definition provided by the HHS and the CDC [21] (used in 5 studies), or at least 12 weeks after COVID-19 diagnosis, consistent with the WHO definition [22] (used in 4 studies). One study assumed long COVID if symptoms were present 6 months after hospital discharge. Two studies did not provide a minimum duration of symptoms following COVID-19 diagnosis to define the condition.

Studies that used a coding-based definition most often defined long COVID as the presence of ICD-10 codes B94.8 (3 studies), U07.1 (2 studies), and U09.9 (2 studies). Individual studies also included ICD-10 codes B97.29, J12.82, M35.8, M35.81, M35.89, and Z86.16. Some studies included other classification codes (PCC diagnosis categories, SNOMED-CT codes).

Lo et al. used a definition of long COVID that was neither symptom- nor coding-based. The study did not use a pre-defined list of post-COVID-19 conditions; instead, post-COVID-19 conditions were identified by a controlled before-after technique as health conditions that newly occurred in COVID-19 cases after the acute infection but did not occur in controls [26].

In studies that did not explicitly refer to long COVID, HCRU, and costs associated with SARS-CoV-2 infection were typically assessed at least a month after COVID-19 diagnosis (9 studies).

### 3.3. Healthcare Resource Use

HCRU was reported in 36 studies, which most often provided data on the use of inpatient care (27 studies), outpatient visits (20 studies), and emergency room (ER) visits (19 studies). Few studies provided information on the utilization of telemedicine, long-term care, home care, and pharmaceutical prescriptions. Data in subsequent paragraphs are presented separately for studies reporting on individuals diagnosed with long COVID and for studies that reported long-term HCRU following COVID-19 diagnosis but did not specifically refer to long COVID. HCRU data from the included studies are summarized in Table 2, with full results presented in Supplementary Table S5.

#### 3.3.1. Overall Long-Term Healthcare Resource Use Following COVID-19

##### Long COVID

In an Australian study, 38.7% of individuals with long COVID sought healthcare services because of ongoing symptoms 3 months following the diagnosis of COVID-19 [23]. Receiving more vaccine doses was protective against developing long COVID, with the risk reduced by up to 60%, but it did not affect HCRU in individuals who did develop long COVID [23]. Controlled studies further demonstrated the long-term HCRU burden associated with long COVID. For up to 2 years after COVID-19 diagnosis, individuals diagnosed with long COVID demonstrated significantly elevated HCRU compared to those with a diagnosis of COVID-19 but not long COVID [27,28] and those who had never been diagnosed with COVID-19 [28,29]; the increase in HCRU in individuals with long COVID in a UK study was over 3-fold (relative risk: 3.28, 95% confidence interval [CI]: 2.54–4.26) [27]. The presence of chronic symptoms persisting at 7 and 15 months of follow-up among individuals with long COVID was associated with further increases in HCRU compared to individuals with long COVID but shorter symptom duration [28].

##### Follow-Up Post-COVID-19 Acute Phase

Prolonged HCRU related to COVID-19 was shown in non-comparative studies conducted in Sweden, where 87.8% of individuals admitted with COVID-19 required care at 28–135 days following hospital discharge and 27.1% of the appointments were COVID-related [40], and in Switzerland, where 40% of individuals required care for reasons related to COVID-19 over approximately 7 months from diagnosis [41]. Controlled studies conducted in the US and Canada that reported on long-term HCRU following a COVID-19 diagnosis demonstrated statistically significant 4–10% increases in long-term total HCRU in individuals with a record of COVID-19 diagnosis compared to controls without a recorded diagnosis of COVID-19 [30,31]. The increase in HCRU after COVID-19 diagnosis was also evident in pre-post studies conducted in the US and Brunei Darussalam, which reported that compared to the 6–12-month period before COVID-19 diagnosis, overall HCRU increased 53–153.5% within 6–12 months following diagnosis [32,33].

##### Factors Associated with Increased HCRU

**Figure 2:**
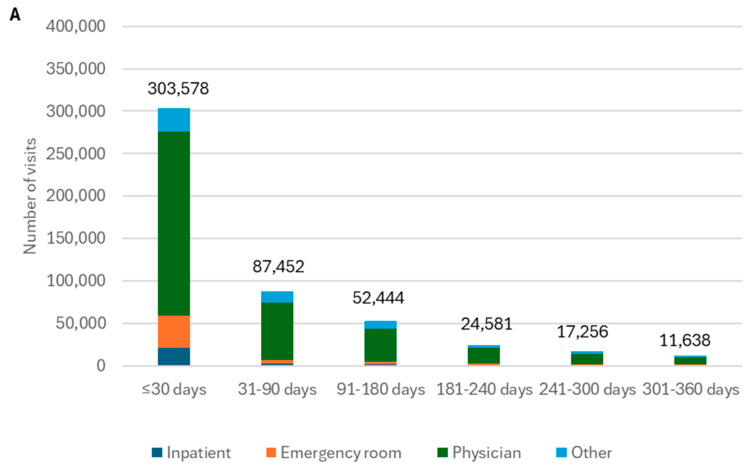

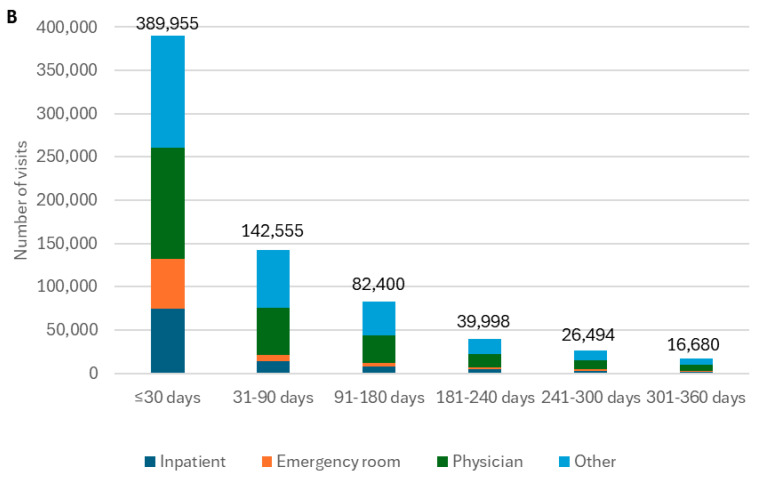
Estimated number of visits across six time intervals spanning 360 days, n = 1,000,000 (Melnick 2023, US [43]). Panel (**A**): commercially insured adult population aged 18–64 years; panel (**B**): Medicare Advantage population aged ≥ 65 years.

Long-term HCRU increased in individuals who were hospitalized during the acute COVID-19 phase and those who were older. In the UK, individuals hospitalized during the acute COVID-19 phase utilized more healthcare services compared with those who were not hospitalized, with a 2.7-fold difference in rates per 100,000 person-weeks between these two groups [42]. Increasing age was another factor associated with HCRU intensification. In a large US claims analysis, excess HCRU associated with COVID-19 was most pronounced in individuals aged 45–64 and ≥65 years compared to younger age groups [32]. Time since COVID-19 diagnosis was another factor that substantially affected HCRU. In general, HCRU declined over time [35,43], with the study by Melnick et al. providing an example of this reduction over time in both the younger, commercially insured population aged 18–64 (Figure 2A) and the Medicare Advantage population aged ≥ 65 (Figure 2B) [43]. The older Medicare Advantage population showed consistently higher HCRU across all time periods compared with commercially insured individuals [43].

#### 3.3.2. Inpatient Admissions

##### Long COVID

Findings on inpatient care utilization in individuals with long COVID were not fully conclusive. In the UK, hospitalization rates were higher in individuals with long COVID compared with three matched control populations of those without long COVID, without COVID-19 diagnosis history, and pre-pandemic [29]. However, in a study in Sweden, a difference-in-difference analysis detected no statistically significant differences in inpatient care utilization between individuals with long COVID and matched controls [34]. Interestingly, in a large US study individuals with long COVID were significantly more likely to have an all-cause hospitalization than those with long flu during 2 months of follow-up (31.9% vs. 26.8%, adjusted odds ratio: 1.06, 95% CI: 1.05–1.08) [24].

##### Follow-Up Post-COVID-19 Acute Phase

The findings regarding long-term utilization of inpatient services following SARS-CoV-2 infection or COVID-19 were mixed in studies that did not specifically refer to long COVID. In single-arm studies, up to 6.7% of individuals were hospitalized within 3–6 months following COVID-19 diagnosis [44,45,46]. In several comparative studies, the utilization of inpatient services by individuals with a record of COVID-19 diagnosis was higher compared to controls without COVID-19 [30,35,39]; however, one US-based study reported no significant differences in the use of inpatient services between individuals with and without a COVID-19 diagnosis over 6 months of follow-up [31].

##### Factors Associated with Increased Utilization of Inpatient Services

High readmission rates after initial hospitalization with COVID-19 were observed in single-arm studies. A Swiss study reported that 10% of individuals hospitalized with COVID-19 were readmitted due to persistent symptoms or complications within approximately 7 months [41]. Over a longer, 12-month period (calendar year 2021) in Germany, the prevalence of hospital admission with a diagnosis code for long COVID was estimated at 5.5% among individuals initially admitted with COVID-19, with 17.2% of the long COVID admissions resulting in an ICU stay [47]. In comparative studies, individuals with a history of hospitalization or ICU admission during their acute COVID-19 phase had increased rates of hospitalization in the 6 months following infection compared with those without COVID-19 [26,36] and the general population [37]. Among individuals initially hospitalized for COVID-19, the all-cause hospital readmission rate was 2.1 and 3.5 times greater compared with the general population during a mean follow-up of approximately 10 and 5 months, respectively [37,38]. Furthermore, in a US-based pre-post study, compared to the 6-month period before diagnosis, a 227% increase in inpatient service utilization due to COVID-19 was demonstrated in the 6 months after diagnosis [32].

#### 3.3.3. Emergency Room Visits

##### Long COVID

Long COVID was associated with high utilization of ER services. In a Swiss study, individuals with long COVID had an increased frequency of ER visits for up to 15 months from COVID-19 diagnosis compared with two control groups (with a COVID-19 diagnosis but no long COVID and without a COVID-19 diagnosis) [28]. The increase in ER utilization was particularly pronounced in those individuals with long COVID whose symptoms persisted for up to 15 months, in whom ER utilization was approximately doubled compared to the two control groups without long COVID and without a COVID-19 diagnosis [28]. Similarly, a study conducted in the UK reported that individuals with long COVID had significantly increased ER attendance compared to non-COVID, pre-long COVID, and pre-pandemic controls, but not compared to those who had a COVID-19 diagnosis but no long COVID over two years of follow-up [29].

##### Follow-Up Post-COVID-19 Acute Phase

Results from single-arm studies that did not specifically refer to long COVID supported the substantial and prolonged burden that COVID-19 poses on emergency services. In Romania, 24% of ER visits occurred later than 3 months from the initial COVID-19 diagnosis [48], while in Italy, 10.6% of individuals admitted to hospital with COVID-19 visited the ER within 12 months of discharge [44].

In comparative studies, up to 36% higher long-term ER utilization over approximately 5–8 months of follow-up was reported in individuals with a COVID-19 diagnosis compared to controls without the diagnosis [30,31,35,39]. The trend was similar in a US pre-post study which reported that a 149% increase in ER utilization due to COVID-19 was observed within 6 months post-COVID-19 diagnosis compared with a pre-diagnosis period of the same length [32]. Factors associated with increased ER utilization were not extensively studied; however, a large US claims analysis conducted by Koumpias et al. reported that males had greater use of emergency services following COVID-19 diagnosis than females [32].

#### 3.3.4. Outpatient Visits

##### Long COVID

In a single-arm Australian study, 38.2% of individuals with long COVID required general practitioner (GP) services because of ongoing symptoms 3 months post-diagnosis of COVID-19 [23]. In comparative studies, there was a clear trend for significantly increased outpatient care utilization in individuals with long COVID. Studies conducted in Switzerland [28], the UK [29], Sweden [34], and Germany [49] reported increased utilization of outpatient services (GP and/or specialist visits) in individuals with long COVID for up to 15 months compared to various control groups, including individuals without a COVID-19 diagnosis and those who had a diagnosis of COVID-19 but not long COVID.

##### Follow-Up Post-COVID-19 Acute Phase

Studies that did not specifically refer to long COVID reported mixed findings regarding long-term utilization of outpatient services in individuals with a COVID-19 diagnosis compared to controls without the diagnosis. Results from single-arm studies indicated high utilization of outpatient services at least 3 months from COVID-19 diagnosis, with 10–35.6% of individuals requiring an outpatient visit for reasons related to COVID-19 [41,46], and 3–7% due to symptoms potentially related to COVID-19 [46]. Similarly, in most comparative studies, those with a record of COVID-19 diagnosis had increased utilization of outpatient services relative to controls [30,35,39]; however, one US study reported significantly lower utilization during 6 months of follow-up (ratio of rate ratio: 0.98, 95% CI: 0.96–0.99) [31].

##### Factors Associated with Increased Utilization of Outpatient Services

Utilization of outpatient care tended to decrease over time following COVID-19 diagnosis. Results from a US insurance claims analysis revealed that primary care was the leading visit type, peaking at 58% within the first 90 days post-diagnosis and remaining high (~48%) for up to 275 days in individuals hospitalized during the acute phase of COVID-19 [50]. Meanwhile, the number of cardiology, pulmonary, endocrinology, and neurology visits remained relatively stable during the same periods, with rates not exceeding 8% [50]. The proportion of individuals with outpatient visits in another US insurance claims analysis was 82.6% in the first month after COVID-19 diagnosis and decreased to 35.4% in month 6, a level similar to that observed in the pre-pandemic control group [35].

Studies conducted in Switzerland, Italy, and Canada reported that individuals who were hospitalized [26,41] or treated in the ICU [36] during the acute phase of COVID-19 had increased outpatient care utilization after discharge. However, results from a UK-based study conducted in the setting of a dedicated post-COVID-19 clinic did not align with this trend and reported that all groups (hospitalized, non-hospitalized, and attended ER during acute infection) had similar rates of onward specialist referral [51].

#### 3.3.5. Utilization of Other Healthcare Services Post-COVID-19 Acute Phase

A retrospective cohort study conducted in Canada was the only one to report on the utilization of long-term care and home care [30]. Long-term care utilization following COVID-19 was significantly higher in individuals with a diagnosis of COVID-19 compared to matched controls without the diagnosis (rate ratio: 2.23, 95% CI: 2.01–2.48); however, this was not the case for home care utilization (rate ratio: 0.99, 95% CI: 0.95–1.03) [30].

Telemedicine (virtual care) utilization after the 2-week acute illness phase was reported in one study conducted in the US and was significantly higher in individuals who had a COVID-19 diagnosis compared to matched controls without a diagnosis of COVID-19 (ratios of rate ratio: 1.14, 95% CI: 1.12–1.16) [31].

Utilization of prescription medicines was reported in one UK-based cohort study [42]. Over a median follow-up of approximately 2 months after COVID-19 diagnosis, prescription of medications was more frequent in individuals hospitalized for COVID-19 compared with those who were not hospitalized [42]. Paracetamol and opioids, respectively, were prescribed 16 and 4.6 times more frequently in hospitalized than in non-hospitalized individuals [42].

### 3.4. Healthcare Costs

Similar to HCRU, costs are reported separately for studies that referred to long COVID and those that reported long-term healthcare costs following SARS-CoV-2 infection but did not specifically mention long COVID. Total healthcare costs are summarized in Table 3, with a further breakdown by cost type provided in Table 4. Detailed results from individual studies are available in Supplementary Table S6.

#### 3.4.1. Total Costs

##### Long COVID

Individuals with long COVID incurred higher total healthcare costs compared with those who had a diagnosis of COVID-19 but not long COVID. In the UK, the mean total cost of care for individuals with long COVID was £3335 per person per year, which was nearly four times higher than the cost of care for the same individuals before the pandemic and nearly three times higher than for matched controls without a COVID-19 diagnosis before and during the pandemic [29]. In Israel, the relative adjusted increase in mean total monthly healthcare costs among individuals with long COVID compared to individuals without a long COVID diagnosis was 1.74, 1.72, and 1.89 at 4, 6, and 12 months after COVID-19 infection, respectively [25]. In the US, all-cause and disease-specific costs of care in individuals with severe or critical COVID-19 who experienced long COVID were 4-fold higher than in those without long COVID, and this was observed for both commercial and Medicare beneficiaries [55].

##### Follow-Up Post-COVID-19 Acute Phase

In studies that did not specifically refer to long COVID, long-term costs following COVID-19 diagnosis in individuals exposed to SARS-CoV-2 were higher than in people without a record of SARS-CoV-2 infection. As assessed by difference-in-difference analysis, there was a 7.6% to 13.1% increase in total healthcare costs per person per month over follow-up ranging from 12 months to up to 15 months. Other studies reported a 1.3- to 2.9-fold relative increase in total healthcare costs compared with controls without a COVID-19 diagnosis over approximately 6 months of follow-up (Table 3). In a pre-post US study, COVID-19 diagnosis was associated with an additional $223.59 in total monthly medical expenditures, corresponding to a 2.75-fold increase in total healthcare costs compared with the pre-diagnosis level [32].

##### Factors Associated with Increased Healthcare Costs

**Figure 3:**
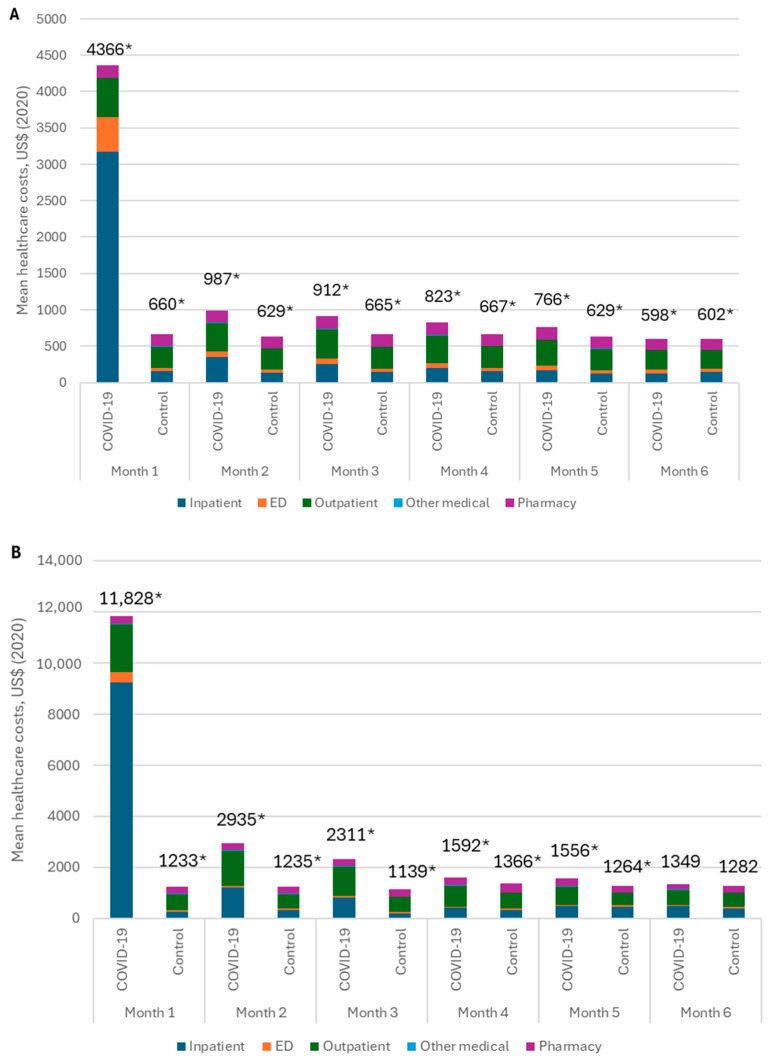
Monthly healthcare costs following COVID-19 diagnosis (COVID-19 group) or the corresponding pre-pandemic period 1 year earlier (matched controls) [35]. Panel (**A**): commercially insured population; panel (**B**): Medicare population. Asterisk marks statistically significant differences at *p* < 0.05. Abbreviations: ED, emergency department.

There was a clear temporal trend, with healthcare costs declining over time from COVID-19 diagnosis. In a US insurance claims analysis, DeMartino reported that individuals with COVID-19 continued to have significantly higher total costs through month 5 compared to matched controls without a COVID-19 diagnosis record, and this was observed in both commercially insured and Medicare populations [35] (Figure 3, panels A and B, respectively). In another US pre-post study, total monthly medical expenditures declined gradually from month to month but remained higher than the pre-diagnosis costs after 6 months of follow-up [32].

Individuals who were hospitalized during the acute phase of SARS-CoV-2 infection incurred up to 10-fold higher healthcare costs after 6 months from diagnosis compared with their non-hospitalized counterparts, a trend observed in two different US claims databases [54,56] and in ex-US studies conducted in Israel [53] and Italy [39].

#### 3.4.2. Inpatient Costs

##### Long COVID

Few studies reported on inpatient costs incurred specifically by individuals with long COVID. In Germany in 2021, total cumulative healthcare costs of long COVID were €136,608,719, with a mean cost of €4583 per case [47]. Among individuals with SARS-CoV-2 infection in Israel, those with long COVID incurred increased inpatient costs over 12 months (adjusted hazard ratio [aHR]: 1.98, 95% CI: 1.20–3.28) compared with individuals without a long COVID diagnosis [25].

##### Follow-Up Post-COVID-19 Acute Phase

Findings regarding inpatient costs were mixed in studies that did not specifically refer to long COVID and instead assessed long-term costs following COVID-19 diagnosis. A 14.6% increase in inpatient healthcare spending was observed among US commercially insured individuals, but not Medicare Advantage insured individuals with a diagnosis of COVID-19 compared with controls without the diagnosis during 12 months of follow-up [52]. Similarly, in an Israeli study with a 15-month follow-up, inpatient costs were increased by 20.3% in individuals with a COVID-19 diagnosis relative to controls without the diagnosis [53]. In another US study, mean inpatient costs rapidly declined after the first month following COVID-19 diagnosis, presumably reflecting the resolution of acute illness. Nonetheless, inpatient costs remained higher in individuals with vs. without a COVID-19 diagnosis over 6 months and this difference was significant up to Month 5 for commercially insured individuals [35].

#### 3.4.3. Outpatient Costs

##### Long COVID

The costs of outpatient visits in individuals with long COVID were reported in two studies. In Israel, during a 12-month follow-up, having long COVID was associated with significantly greater expenditure due to physician visits (aHR: 1.98, 95% CI: 1.41–2.78) and lab and imaging tests (aHR: 1.60, 95% CI: 1.44–1.78) [25]. In the UK, compared with controls without diagnosed COVID-19, a long COVID diagnosis was associated with an over 3-fold increase in primary care consultation costs and an incremental cost of £30.50 per person, while reporting symptoms of long COVID (but not having a diagnosis code) was associated with a 6-fold increase in primary care consultation costs and an incremental cost of £57.60 per person during a minimum follow-up of 12 weeks [57]. This study also assessed factors associated with increased outpatient costs. In addition to long COVID diagnosis or symptoms, other factors associated with increased costs of primary care consultations ≥ 12 weeks from COVID-19 diagnosis were increasing age, female sex, being obese or having a missing BMI record, increasing comorbidity burden, and more prior consultations [57]. Conversely, Black individuals had 6% lower costs compared to White individuals; however, no significant differences were observed for other ethnic groups [57]. The relationship between socioeconomic status and primary care consultation costs was mixed and did not follow a clear gradient [57].

##### Follow-Up Post-COVID-19 Acute Phase

High long-term outpatient spending following a COVID-19 diagnosis was evident in a single-arm study conducted in Sweden, where the total costs of outpatient visits for all study patients (n = 466) amounted to €77,311.30 over 6 months of follow-up following hospitalization for COVID-19 [40]. In comparative studies that assessed long-term outpatient costs after a COVID-19 diagnosis but did not specifically refer to long COVID, the costs were substantially higher in individuals with COVID-19 compared to those with no documented COVID-19. This was evident across two US-based studies which assessed outpatient costs in individuals with a record of COVID-19 diagnosis compared with controls without the diagnosis among commercial and Medicare Advantage beneficiaries, with cost increases of up to 14.8% and 44.6% observed in the two populations, respectively, over a follow-up of 12 months [35,52]. Similar results were obtained in a study conducted in Israel, in which higher excess costs in the population with a COVID-19 diagnosis were observed compared to control population without the diagnosis for primary care (+7.5%), medical specialist visits (+8.0%), paramedical professions visits (+8.0%), ER visits (+6.8%), and ambulatory care visits (+8.4%) [53].

#### 3.4.4. Costs of Prescription Medication

##### Long COVID

In individuals diagnosed with long COVID in Israel, medication costs were numerically higher compared with controls without long COVID over 12 months of follow-up, but the difference was not statistically significant [25].

##### Follow-Up Post-COVID-19 Acute Phase

Three studies reported long-term costs of prescription medication after a COVID-19 diagnosis but did not specifically refer to long COVID. Their findings were inconclusive. A modest, approximately 6% decrease in medication costs compared with controls without COVID-19 was observed among individuals with a COVID-19 diagnosis in the US (commercially insured individuals) and Israel [52,53]. Among the older Medicare Advantage population in the aforementioned US study, individuals with a COVID-19 diagnosis experienced a 19% increase in pharmacy costs compared with controls [52].

### 3.5. Pediatric Studies

There were only three studies that evaluated long-term HCRU and costs of COVID-19 in children; results from these studies are inconclusive. One study reported no HCRU increase post-COVID-19 and a slight but statistically significant decrease in total healthcare and pharmacy costs in individuals with a diagnosis of COVID-19 compared to controls without the diagnosis [58]. Another study reported a 1.75-fold increase in direct medical costs for children with a diagnosis of COVID-19 [54].

## 4. Discussion

This review comprehensively described the HCRU and related costs associated with long COVID or, where this was not specifically considered, long-term outcomes following a COVID-19 diagnosis. In the studies that specifically considered individuals with long COVID, HCRU was significantly elevated for up to 2 years after COVID-19 diagnosis compared not only to individuals with no record of a COVID-19 diagnosis but also to those who had COVID-19 but did not have long COVID [27,28,29]. This pattern was observed across a spectrum of healthcare resources, including overall HCRU [27,28], inpatient [29], emergency [28,29], and outpatient services [28,29], suggesting a substantial excess burden posed on the healthcare systems by long COVID. In studies that described follow-up after the acute COVID-19 phase but did not specifically refer to long COVID, individuals with a COVID-19 diagnosis required more healthcare resources than those without a COVID-19 diagnosis even months after the acute infection phase subsided, and this was evident across various services including ER visits, inpatient, and outpatient encounters. Although the highest demands for healthcare services were generally reported in the first month following a COVID-19 diagnosis and gradually decreased in the subsequent months, demand consistently remained at an increased level compared to controls without a documented SARS-CoV-2 infection for at least 6 months after diagnosis. Similarly, pre-post-COVID-19 diagnosis studies reported increased HCRU in the post-diagnosis period.

High long-term HCRU among individuals with a COVID-19 diagnosis led to increased healthcare costs compared with controls without the diagnosis; total healthcare costs were up to 3-fold higher in individuals with a COVID-19 diagnosis compared to controls without the diagnosis over 6 months. In studies that specifically reported on long COVID, individuals with long COVID had increased HCRU and associated costs not only when compared with those without a COVID diagnosis, but also those who had been infected with SARS-CoV-2 but did not develop long COVID. The increased burden was still observed 2 years after diagnosis and the impact of long COVID on healthcare utilization was greater than that of long flu.

Risk factors for higher than usual HCRU and costs were not the focus of this review; however, the available data suggest that having severe acute COVID-19 disease (requiring hospitalization with or without ICU admission) was a risk factor for increased long-term HCRU and costs compared with having milder forms of the disease [36,39,41,42,53,54,56]. Having long-term persistent long COVID symptoms was associated with increases in HCRU compared to individuals with long COVID but shorter symptom duration, and this was particularly pronounced in patients with very chronic symptoms persisting for up to 15 months [28]. Other risk factors for elevated HCRU and costs included older age [26,32,39,41,42,46,48], comorbidities [39,56,57,59], female sex [39,57], and the lack of anti-SARS-CoV-2 vaccination [39].

The major strength of this review was the comprehensive approach utilized to capture HCRU and costs that included preprints and conference abstracts, both of which enable the relevant data to be shared with the scientific community promptly, a considerable advantage in the setting of a novel condition which long-term sequelae are still being explored. Included studies were assessed in a rigorous manner and the reporting generally followed the PRISMA guidance [16], although no risk of bias or certainty of evidence assessment was performed.

There are also some limitations to this review that should be taken into account when interpreting the presented data. The included studies differed substantially in terms of design, analytical methods, the evaluated population, and geographic locations (and therefore healthcare systems). Moreover, there was notable heterogeneity in the definitions of long COVID which further complicated the interpretation of results across studies and the ability to draw unambiguous conclusions about the economic impact of long COVID. In this context, comprehensive definitions such as the one proposed by the National Academies of Sciences, Engineering, and Medicine (NASEM) and efforts aiming to standardize the definition of long COVID are needed to ensure a common baseline and comparability across studies [9]. Results from studies evaluating long-term HCRU and costs in the pediatric population were scarce and inconclusive. Furthermore, HCRU and costs were assessed only from the healthcare perspective and not the societal perspective; therefore, outcomes such as lost productivity costs or work time lost attending and/or traveling to medical appointments were not included. Considering that in some individuals, the symptoms of long COVID are so severe that affected individuals meet the legal definition of disability [16,60], the exclusion of indirect costs is an important limitation. The included studies relied on a record of COVID-19 diagnosis and therefore did not capture individuals with an asymptomatic or mild infection course and/or those living in remote areas who sought neither testing nor treatment. As asymptomatic and mild acute COVID-19 disease is considered an important contributor to the overall long COVID burden [9], it is likely that a significant portion of individuals with long COVID, and the associated disease burden, were not captured in the included studies because they had no record of an initial COVID-19 diagnosis. Furthermore, careful consideration should be given to the different control groups available in many studies. Comparisons with pre-pandemic controls (self or matched) may be affected by the disruption to the health services caused by the pandemic, while contemporaneous controls without a COVID-19 diagnosis allow for control of this. Finally, the risk of bias in the included studies was not formally assessed, although the studies varied in their apparent quality and depth of reporting. Given the aforementioned limitations, it is likely that this review underestimates the burden of long COVID.

## 5. Conclusions

Long COVID imposes a sustained and significant burden on healthcare systems worldwide, although more high-quality studies are needed to better understand the true economic impact of long COVID, particularly in the pediatric population. Unambiguous evidence on the burden of long COVID is needed to inform and facilitate the development of public health decisions and policies addressing the consequences of COVID-19. In the absence of effective treatments, prioritizing preventive measures against acute COVID-19, such as vaccination, may be crucial for preventing the development of long COVID, thereby mitigating long-term HCRU and healthcare spending.

## Figures and Tables

**Table 1:** Eligibility criteria for publications included in the review.

**Population**	Children and adults diagnosed with long COVID, post-COVID-19 syndrome, or post-acute COVID sequelae/conditions (hereafter referred to as long COVID) Because long COVID is often undiagnosed, studies with long-term follow-up of individuals after their COVID-19 diagnosis were also included, even if the studies did not specifically refer to long COVID
**Outcomes**	Long-term healthcare costs and/or HCRU
**Study and publication type**	Observational studies published as: ○ Peer-reviewed journal articles ○ Preprint journal articles ○ Country-specific reports
**Countries of interest**	No geographic restrictions were applied for PubMed search and hand searches HTA Agencies website search was restricted to European countries (EU4 and the UK), the US, Canada, Australia, Korea, Taiwan, Israel, and Japan
**Language**	English only

Abbreviations: EU4, France, Germany, Italy, Spain; HCRU, healthcare resource use; HTA, health technology assessment.

**Table 2:** Summary of findings on HCRU in patients with long COVID or long-term HCRU in patients with COVID-19.

Key Findings	Study(s)
**Overall**	
**Long-term HCRU in patients with** **long COVID**	
Patients with long COVID had significantly elevated HCRU for up to 2 years after diagnosis vs. patients with COVID-19 without a long COVID diagnosis or without COVID-19 infection	Debski 2022 [27]; Nehme 2022 [28]; Mu 2023 [29]
**Long-term HCRU in patients with COVID-19**	
A significant 4–10% increase in long-term total HCRU was observed in patients with COVID-19 vs. patients without COVID-19 in the United States and Canada.	McNaughton 2022 [30]; Tartof 2022 [31]
Compared to the 6–12-month period before COVID-19 diagnosis, overall HCRU increased 53–153.5% within 6–12 months following diagnosis.	Koumpias 2022 [32]; Abdullah 2023 [33]
**Inpatient Services**	
**Long-term HCRU in patients with** **long COVID**	
Findings on inpatient HCRU in patients with Long COVID were inconclusive. One study reported significant excess inpatient admissions in individuals with long COVID compared to self-controlled pre-COVD-19, individuals with no COVID-19, and the pre-pandemic period. Results from another study indicated no statistically significant differences between individuals with long COVID and matched non-long COVID controls.	Mu 2023 [29]; Hedberg 2022 [34]
Outpatients with long COVID were more likely to have an all-cause hospitalization than patients with Long Flu in a study from the United States.	Fung 2023 [24]
**Long-term HCRU post-COVID-19 acute phase**	
Individuals with a previous SARS-CoV-2 infection had increased rates of hospitalizations compared with patients who did not have a previous SARS-CoV-2 infection.	DeMartino 2022 [35]; Castriotta 2023 [36]; Ayoubkani 2021 [37]; Lo 2023 [26]; Tisler 2022 [38]; Formoso 2023 [39]; McNaughton 2022 [30]
**Outpatient and Emergency Services**	
**Long-term HCRU in patients with** **long COVID**	
A trend for significantly increased outpatient service utilization was observed in patients with long COVID, including an increase in frequency of ED, primary care, and specialist visits for up to 15 months compared with those without long COVID or COVID-19	Nehme 2022 [28]
**Long-term HCRU post-COVID-19 acute phase**	
A significant increase in long-term outpatient and ED services utilization was seen in patients with COVID-19 compared with controls without COVID-19	Tartof 2022 [31]; McNaughton 2022 [30]; Formoso 2023 [39]
Long-term care/home care, virtual care, and pharmaceutical care were higher in COVID-19 patients vs. those without COVID-19	Tartof 2022 [31]; McNaughton 2022 [30]; Formoso 2023 [39]

Abbreviations: ED, emergency department; HCRU, healthcare resource use.

**Table 3:** Long-term total healthcare costs following COVID-19 diagnosis.

Studies Utilizing a DiD Approach vs. Controls Without a COVID-19 Diagnosis						
Study (Country)	Follow-Up	Population	DiD (Per Person Per m) ^a^		Relative Increase in Total Healthcare Costs	
Chambers 2023 (US) [52]	12 m	Overall (Commercial) n = 14,448	$41.61		7.7%	
		Overall (Medicare) n = 3260	$97.30		13.1%	
Wolff Sagy 2023 (Israel) [53]	DiD calculated for a maximum post-recovery follow-up of 15 m (average: 8.25 m, median: 9 m)	Overall n = 1,285,736	$7.6		7.6%	
**Studies reporting relative differences between individuals with and without COVID-19 diagnosis**						
**Study (country)**	**Follow-up**	**Population**	**Difference in total healthcare costs**			
			**AD ^b^**	**RD ^c^**		***p* value**
Pike 2023 (US) [54]	6 months (1-, 3-, and 6 m periods starting 31 d after the index date)	Children n = 58,180	$1011 (6 m)	1.75		<0.001
		Adults n = 987,060	$1562 (6 m)	1.56		<0.001
DeMartino 2022 (US) [35]	6 m (after the index date)	Overall (Commercial) n = 301,462	$763	2.18		<0.001
		Overall (Medicare) n = 3724	$2337	2.85		<0.001
Formoso 2023 (Italy) [39]	Median 152 d (range 1–180 d)	Overall n = 72,072	€61.27	1.27		Not defined

Abbreviations: AD, absolute difference; d, day; DiD, difference-in-difference; m, month; RD, relative difference. Note: ^a^ DiD analysis was used to compare changes in healthcare spending between the 12 months before (baseline period) and the 12 months after (post period) COVID-19 diagnosis for COVID-19 cases and contemporaneous matched controls without COVID-19. ^b^ Costs among individuals with a COVID-19 diagnosis minus costs among controls. ^c^ Costs among individuals with COVID-19 divided by costs among controls.

**Table 4:** Summary of findings on healthcare costs in patients with long COVID or those with long-term costs post-COVID-19 acute phase.

Key Findings	Study(s)
**Overall**	
**Long-term costs in patients with** **long COVID**	
Patients with long COVID incurred higher total healthcare costs compared with patients without long COVID.	Mu 2023 [29]; Tene 2023 [25]; Patterson 2022 [55]
**Long-term costs post-COVID-19 acute phase**	
Long-term total healthcare costs in patients exposed to SARS-CoV-2/ with a COVID-19 diagnosis were higher compared with those without prior SARS-CoV-2 infection/COVID-19 diagnosis.	Formoso 2023 [39]; Chambers 2023 [52]; DeMartino 2022 [35]; Khan 2024 [56]; Pike 2023 [54]; Wolff Sagy 2023 [53]; Koumpias 2022 [32]
**Inpatient costs**	
**Long-term costs in patients with** **long COVID**	
Patients with long COVID incurred increased inpatient costs compared with patients without long COVID.	Tene 2023 [25]
**Long-term costs post-COVID-19 acute phase**	
Compared with controls, US commercially insured individuals with COVID-19 had an increase in inpatient healthcare expenditure over 12 months, while less of an increase in inpatient costs was observed in the Medicare Advantage population with COVID-19 vs. controls. In an Israeli study with a 15-month follow-up, inpatient costs were increased by 20.3% compared with controls.	Chambers 2023 [52]; Wolff Sagy 2023 [53]
**Outpatient and ED Costs**	
**Long-term costs in patients with** **long COVID**	
Patients with long COVID incurred increased outpatient costs due to physician visits, laboratory/imaging procedures, and primary care visit costs compared with patients without long COVID	Tene 2023 [25]; Tufts 2023 [57]
**Long-term costs post-COVID-19 acute phase**	
Long-term outpatient costs in SARS-CoV-2 infected individuals were higher than in those without a prior SARS-CoV-2 infection for commercially insured and Medicare Advantage populations in the United States.	Chambers 2023 [52]; DeMartino 2022 [35]
Higher excess costs in patients with confirmed COVID-19 diagnosis compared to unexposed patients were observed for primary care, medical specialists’ visits, paramedical professions visits, ED visits, and ambulatory care visits.	Wolff Sagy 2023 [53]
**Pharmaceutical Costs**	
**Long-term costs in patients with** **long COVID**	
Medication costs trended higher, although not statistically significant, in patients with long COVID during ae 12-month follow-up compared with patients without long COVID	Tene 2023 [25]
**Long-term costs post-COVID-19 acute phase**	
Compared with controls, a 6% decrease in medication costs was observed in individuals with COVID-19 among the US commercially insured population and in an Israeli cohort study, while a 19% increase vs. controls was observed in COVID-19 patients covered by Medicare Advantage.	Chambers 2023 [52]; Wolff Sagy 2023 [53]
Long-term pharmaceutical costs remained relatively constant over 6 months in SARS-CoV-2 infected patients and were comparable to those without prior SARS-CoV-2 infection	DeMartino 2022 [35]

Abbreviations: ED, emergency department.

## Data Availability

As this was a literature review, no new data were generated during the study.

## Supplementary Materials

The following supporting information can be downloaded at: https://www.mdpi.com/article/10.3390/jmahp13010007/s1, Table S1: PubMed search strategy title; Table S2: List of Health Technology Agencies searched; Table S3: Search queries used in additional searches; Table S4: Definitions of long COVID used in studies that specifically considered HCRU and/or costs associated with long COVID [61,62]; Table S5: Summary of study results on long-term HCRU in patients with long COVID or with persistent symptoms of COVID-19 [63]; Table S6: Summary of study results on long-term healthcare costs in patients with long COVID or with persistent symptoms of COVID-19.
