# Supplementary material for: Healthcare Resource Utilization (HCRU) and Direct Medical Costs Associated with Long COVID or Post-COVID-19 Conditions: Findings from a Literature Review
Source: J Mark Access Health Policy. 2025 Feb 12;13(1):7. doi: 10.3390/jmahp13010007 (PMC11843940; doi:10.3390/jmahp13010007)
Supplement: Supplementary file 1 [file jmahp-13-00007-s001.zip › jmahp-3240519-supplementary_ELU.pdf]

Journal of
Market Access
& Health Policy

MDPI

Supplementary Table S1. PubMed search strategy.

No	Query
#1	Long-covid[tw]
#2	Long-haul COVID-19[tw]
#3	Long-haul COVID19[tw]
#4	Long COVID-19[tw]
#5	Long Covid19[tw]
#6	Chronic covid[tw]
#7	Post-covid19[tw]
#8	Post-covid-19[tw]
#9	Post-acute covid[tw]
#10	Post-acute covid-19[tw]
#11	Post-acute covid19[tw]
#12	Postacute covid[tw]
#13	Postacute covid19[tw]
#14	Postacute covid-19[tw]
#15	Post-acute sequelae of COVID-19[tw]
#16	Postacute sequelae of COVID-19[tw]
#17	Postacute sequelae of COVID[tw]
#18	Post-acute sequelae of COVID[tw]
#19	Post-acute coronavirus syndrome[tw]
#20	Postacute coronavirus syndrome[tw]
#21	Post-Acute COVID-19 Syndrome[tw]
#22	Postacute COVID-19 Syndrome[tw]
#23	Postacute COVID Syndrome[tw]
#24	Post-Acute COVID Syndrome[tw]
#25	Post-acute COVID sequelae[tw]
#26	Post-acute COVID19 sequelae[tw]
#27	Post-acute COVID-19 sequelae[tw]
#28	Post-covid condition*[tw]
#29	Post-covid-19 condition*[tw]
#30	Post-covid19 condition*[tw]
#31	Chronic covid syndrome[tw]
#32	Chronic covid19 syndrome[tw]
#33	Chronic covid-19 syndrome[tw]
#34	Long-term covid syndrome[tw]
#35	Long-term covid19 syndrome[tw]
#36	Long-term covid-19 syndrome[tw]
#37	PASC[tiab]
#38	Post-Acute COVID-19 Syndrome[MESH]
#39	#1 OR #2 OR #3 OR #4 OR #5 OR #6 OR #7 OR #8 OR #9 OR #10 OR #11 OR #12 OR #13 OR #14 OR #15 OR #16 OR #17 OR #18 OR #19 OR #20 OR #21 OR #22 OR #23 OR #24 OR #25 OR #26 OR #27 OR #28 OR #29 OR #30 OR #31 OR #32 OR #33 OR #34 OR #35 OR #36 OR #37 OR #38
#40	COVID[tiab]
#41	COVID-19[tiab]
#42	COVID19[tiab]
#43	SARS-CoV-2[tiab]
#44	SARSCoV2[tiab]
#45	2019-nCoV[tiab]
#46	Coronavirus[tiab]

#47	Severe Acute Respiratory Syndrome Coronavirus[tiab]
#48	#40 OR #41 OR #42 OR #43 OR #44 OR #45 OR #46 OR #47
#49	Comorbidit* [tiab]
#50	Complication*[tiab]
#51	Consequence*[tiab]
#52	Implication* [tiab]
#53	Late effect*[tiab]
#54	Sequelae[tiab]
#55	#49 OR #50 OR #51 OR #52 OR #53 OR #54
#56	#48 AND #55
#57	long-term[tiab]
#58	longitudinal[tiab]
#59	follow-up[tiab]
#60	post-acute[tiab]
#61	post-discharge[tiab]
#62	#57 OR #58 OR #59 OR #60 OR #61
#63	#56 AND #62
#64	#39 OR #63
#65	Medical Expenditure*[tw]
#66	Healthcare Utilization[tw]
#67	Healthcare Utilisation[tw]
#68	Health Care Cost*[tw]
#69	Healthcare cost*[tw]
#70	Health policy[tiab]
#71	Health resource utilization[tw]
#72	Health resource utilisation[tw]
#73	HCRU[tiab]
#74	health economics[tw]
#75	health services research[tw]
#76	Medical cost*[tw]
#77	Cost*[tiab]
#78	Economic burden[tiab]
#79	Medical spending*[tw]
#80	Cost of illness[tw]
#81	Hospitalization cost*[tw]
#82	Hospitalisation cost*[tw]
#83	Hospital cost*[tw]
#84	Spending[tiab]
#85	Health service cost[tw]
#86	economic*[tiab]
#87	cost effectiveness[tw]
#88	Health Care Costs[MESH]
#89	Health Expenditures[MESH]
#90	Health Resources[MESH]
#91	Health Care Utilization[MESH]
#92	Medicare*[MESH]
#93	Hospital costs[MESH]
#94	#65 OR #66 OR #67 OR #68 OR #69 OR #70 OR #71 OR #72 OR #73 OR #74 OR #75 OR #76 OR #77 OR #78 OR #79 OR #80 OR #81 OR #82 OR #83 OR #84 OR #85 OR #86 OR #87 OR #88 OR #89 OR #90 OR #91 OR #92 OR #93
#95	#64 AND #94

Supplementary Table S2. List of Health Technology Agencies searched.

**Country**	**Health Technology Agency**
International Organizations	International Network of Agencies for Health Technology Assessment; (INAHTA), World Health Organization, Our World in Data
UK	National Institute for Health and Care Excellence (NICE), Health Technology Wales (HTW), Scottish Medicines Consortium (SMC), All Wales Medicines Strategy Group (AWMSG), National Institute for Health Research (NIHR), Health Information and Quality Authority (HIQA)
France	Haute Autorité de Santé (HAS)
Germany	Gemeinsame Bundesausschuss (G-BA), Institute for Quality and Efficiency in Health Care (IQWIG), German HTA Agency at the German Institute for Medical Documentation and Information (DAHTA at DIMDI), Robert Koch Institute (RKI)
Spain	Agencia Española de Medicamentos y Productos Sanitarios (AEMPS), IInstituto de Salud Carlos III - The Carlos III Health Institute (ISCIII), Andalusian HTA Agency (AETSA), Galician HTA Agency (AVALIA-T), Catalan Agency for Health Information, Assessment and Quality (AQuAS), HTA Unit in Madrid (UETS-Madrit), Basque HTA Office (OSTEBA)
Italy	Italian Medicines Agency (it. Agenzia Italiana Del Farmaco, AIFA), Regional coordination for medicines (CRUF), Regional Health Agency in Emilia Romagna (ER Salute), Regional Technical and Administrative Support Body of the Tuscan Health (ESTAR)
Korea	National Evidence-based Healthcare Collaborating Agency (NECA)
Taiwan	Center for Drug Evaluation (CDE)
Japan	Center for Outcomes Research and Economic Evaluation for Health in Japan (C2H)
Australia	Pharmaceutical Benefits Advisory Committee (PBAC), Adelaide Health Technology Assessment (AHTA)
US	Agency for Healthcare Research and Quality (AHRQ), Institute for Clinical and Economic Review (ICER)
Canada	Canadian Agency for Drugs and Technologies in Health (CADTH)
Israel	Israeli Center for Technology Assessment in Health Care (ICTAHC)

Supplementary Table S3. Search queries used in additional searches.

Source	Search terms
Google Scholar	• healthcare cost "long covid"<br>• healthcare cost "post covid”<br>• healthcare cost "post covid syndrome”<br>• healthcare utilisation "long covid“<br>• healthcare utilisation "post covid syndrome“<br>• healthcare utilization "long covid”<br>• healthcare utilization "post covid syndrome"
medRxiv and SSRN	• One of the following: “long covid”, “post covid”, ”post acute COVID sequelae” “sequelae of covid”,<br>• AND one of the following: “cost”, “utilisation”, “utilization”, “HCRU”, “economic”, “expenditure”, “spending”

Supplementary Table S4. Definitions of long COVID used in studies that specifically considered HCRU and/or costs associated with long COVID.

Study	Definition
**Symptom-based definitions (12 studies)**	
Abdullah 2023 [33]	A condition occurring usually 3 months from the onset of COVID-19 with symptoms that last for at least 2 months that cannot be explained by an alternative diagnosis
Debski 2022 [27]	One or more new symptoms (e.g., post-COVID breathlessness, use of any health services in relation to post-COVID syndrome, chest pain)
Fung 2023 [24]	Any of the 11 symptoms identified in the WHO definition from 1 to 3 months post-infection (e.g., fatigue/malaise/weakness, muscle/joint pain)
Gil 2023 [61]	Symptoms assessed 6 months after discharge from the hospital, e.g., fatigue, myalgia, dyspnea, or headache
Kirchberger 2023 [49]	Presence of fatigue, dyspnea on exertion, memory problems, or concentration problems, either at the baseline assessment (median 9 months after acute infection) or during follow-up (median 26 months after acute infection)
Menges 2021 [41]	Four-category scale (feeling “recovered and symptom-free”, “better but not fully recovered”, “neither better nor worse”, or “worse”) for accessing the individual's recovery more than 4 weeks after the acute COVID-19 phase
Miller 2023 [59]	Following the individual’s first positive test, one or more of 17 symptoms that lasted >4 weeks since they first experienced the symptom(s) (e.g., change in mood, change in smell or taste, chest pain/pressure, cough)
Nehme 2022 [28]	Having symptoms for more than 12 weeks after the infection (e.g., fatigue, headache, change in smell, mental exhaustion)
Patterson 2022 [55]	Duration of disease exceeding 12 weeks
Tene 2023 [25]	Probable long COVID: Individuals with physician-diagnosed post-COVID symptoms at more than 4 weeks from the first positive RT-PCR, even if they did not have a specific long COVID diagnosis code in their electronic medical record
Tufts 2023 [57]	Symptoms persisting beyond 12 weeks of infection in individuals with a confirmed SARS-CoV-2 infection
Woldegiorgis 2023 [23]	New or ongoing COVID-19 illness-related symptoms or health issues 90 days post-diagnosis
**Coding-based definitions (8 studies)**	
Encinosa 2023 [62]	ICD-10: Z86.16; ICD-10: U07.1 + B94.8 for COVID-19 sequelae

Fung 2023 [24]	ICD-10: B94.8
Hedberg 2022 [34]	ICD-10: U09.9
Melnick 2023 [43]	ICD-10: B97.29, U07.1, J12.82, B94.8, and M35.89 for identification of individuals with subsequent COVID-19-related healthcare utilization<br><br>ICD-10: M35.8 and M35.81 were also indicative of sequelae of a COVID-19 diagnosis if code B94.8 was on the same claim line
Mu 2023 [29]	20 SNOMED-CT codes (e.g., 1325161000000102 Post-COVID-19 syndrome); ICD-10: U074
Tartof 2022 [31]	44 post-COVID condition (PCC) diagnosis categories, e.g., abdominal pain (R10), cough (R05) or constitutional fever/malaise/fatigue (R50, R61, R53, G93.3)
Tene 2023 [25]	ICD diagnosis code of post-COVID-19 conditions (not specified)
Walter 2022 [47]	ICD-10: U09.9
**Other definitions (1 study)**	
Lo 2023 [26]	Post-COVID-19 conditions were identified by a controlled before-after technique instead of a pre-defined list of conditions. Disease classes up to 3 years before the index date ("before") and during the entire follow-up ("after") period were examined for cases and matched controls; new disease classes (in the "after" period) between the case and control groups were compared

Abbreviations: ICD, International Classification of Diseases; RT-PCR, reverse transcriptase-polymerase chain reaction; WHO, World Health Organization. Note: The following ICD codes appear in the long COVID definitions: B94.8 Sequelae of other specified infectious and parasitic diseases; B97.29 Other coronavirus as the cause of diseases classified elsewhere; J12.82 Pneumonia due to coronavirus disease 2019; M35.8 Other specified systemic involvement of connective tissue; M35.81 Multisystem inflammatory syndrome; M35.89 Other specified systemic involvement of connective tissue; U07.1 COVID-19; U074 Post-COVID-19 condition (based on study; no code in current ICD-10 version); U09.9 Post COVID-19 condition, unspecified; Z86.16 Personal history of COVID-19.

Supplementary Table S5. Summary of study results on long-term HCRU in patients with long COVID or with persistent symptoms of COVID-19

Study	Study arm(s)	Follow-up	Results
**Overall**			
**Long COVID**			
Debski 2022 [27]	• Patients with post-COVID syndrome	NA	Overall, patients with post-COVID-19 symptoms had an increased probability of using health services compared with those without symptoms (RR = 3.28, 95% CI: 2.54–4.26; $p < 0.05$).
Woldegiorgis 2023 [23]	• Long COVID	3 months	Over 38.7% of patients with long COVID sought healthcare services because of ongoing symptoms 3 months post-diagnosis of COVID-19; 38.2% scheduled a GP appointment, 3.9% presented to ED, and 1.6% were admitted to hospital.
Nehme 2022 [28]	• Post-COVID condition with chronification of symptoms;<br>• Post-COVID condition without chronification of symptoms;<br>• COVID-19 without post-COVID condition;<br>• No COVID-19	15 months	Symptom chronicity (lasting up to 15 months post-infection) led to increased overall healthcare service utilization (62.2%) compared with individuals with post-COVID-19 without persistent symptoms (43.6%), individuals without post-COVID-19 (27.9%), and those without positive SARS-CoV-2 test results (31.6%).
**Long-term HCRU in patients with COVID-19**			
Ashman 2023 [40]	• COVID-19 (hospitalized)	6 months	87.8% of patients required care at 28-135 days post-hospital discharge.<br>27.1% of appointments involved COVID-19 diagnosis, among which most (56.1%) were with physiotherapists.
Menges 2021 [41]	• COVID-19	7.2 months	40% of patients required care for reasons related to COVID-19, and most went to GPs (36%). 10% of hospitalized patients needed rehospitalization.
Melnick 2023 [43]	• COVID-19	12 months	The highest number of healthcare visits was observed in the first 30 days post-COVID-19 diagnosis, with continued care still needed beyond the first month. Medicare Advantage patients consistently showed higher utilization than commercially insured adults, resulting in an estimated 1.2 million healthcare visits over 360 days in the total population of 2 million patients.
Koumpias 2022 [32]	• Self-controlled study: pre and post COVID-19 diagnosis	6 months before and after COVID-19 diagnosis	A COVID-19 diagnosis correlated with a 153% increase in the average number of healthcare visits per month over the 6 months following diagnosis compared to a 6-month period before COVID-19 diagnosis (mean 1.21 vs. 0.48 number of services).
Abdullah 2023 [33]	• Self-controlled study: pre and post COVID-19 diagnosis	12 months before and after	A notable increase (+53%) in overall healthcare visits was observed during the 12 months following COVID-19 recovery compared to the preceding 12-month period (mean 5.0 vs 3.2 visits; $p<0.001$).

Study	Study arm(s)	Follow-up	Results
		COVID-19 diagnosis	
Tartof 2022 [31]	• Positive SARS-CoV-2 tests results<br>• Negative SARS-CoV-2 test results	6 months	Difference-in-difference analyses demonstrated an overall increase of 4% in healthcare utilization over 6 months among patients with positive test results for SARS-CoV-2 vs. negative results (RRR=1.04, 95% CI: 1.03-1.05). By setting, COVID-19-associated increases in healthcare utilization were highest for virtual encounters (RRR=1.14, 95% CI: 1.12-1.16), followed by ED encounters (RRR=1.08, 95% CI: 1.04-1.12); outpatient encounters declined slightly (RRR=0.98, 95% CI: 0.96-0.99). An additional 27,217 healthcare visits were attributed to COVID-19 over 6 months (212.9, 95%CI: 146.5-278.4 visits per 1000 patients).
McNaughton 2022 [30]	• Positive SARS-CoV-2 tests results<br>• Negative SARS-CoV-2 test results	Mean 240 days	During the mean 240 days of follow-up, patients with SARS-CoV-2 infection had 64.48 additional healthcare encounters at the 99th percentile of the per-person-year rate compared with patients with negative SARS-CoV-2 test results. Rate ratios for the test-positive vs. test-negative comparison were statistically significant (rate ratio 1.10, 95%CI: 1.08-1.2).
**Inpatient Services**			
**Long COVID**			
Walter 2022 [47]	• Long COVID	NA	Among German patients hospitalized with a long COVID diagnosis, as many as 17.2% required ICU treatment.
Hedberg 2022 [34]	• Long COVID;<br>• No long COVID	4-12 months after COVID-19 diagnosis	For inpatient care, no clear trend was observed after (vs. before) the acute infection in the three severity groups (non-hospitalized, hospitalized, and ICU-treated). There were no statistically significant differences between groups assessed by difference-in-difference analysis.
Mu 2023 [29]	• Long COVID;<br>• Pre-pandemic (self-controlled);<br>• COVID only, no long COVID;<br>• Unaffected by pandemic (before Dec 21, 2019;<br>• Contemporary non-COVID	Mean from 395 to 780 days depending on study arm	Hospitalization rates were higher in patients with long COVID compared with patients without long COVID, and without COVID-19, and compared to the corresponding pre-pandemic period (p<0.001) but not to the COVID only, no long COVID group.
Fung 2023 [24]	• Long COVID;<br>• Long flu	2 months	Outpatients with long COVID were more likely to have any-cause hospitalization than patients with long flu (31.9% vs. 26.8%, adjusted OR=1.06, 95%CI: 1.05-1.08, p<0.001).
**Long-term HCRU in patients with COVID-19**			
Comelli 2022 [44]	• COVID-19 (hospitalized)	12 months	19.4% of patients needed severe medical care post-hospital discharge, with 10.6% visiting the ED and 6.6% requiring hospitalization.
Griffin 2023 [45]	• COVID-19	3 months	Hospitalization rates were 6.7% within the initial 90 days, with a peak of 5.6% observed within the initial 30 days.

Study	Study arm(s)	Follow-up	Results
Nica 2022 [48]	• COVID-19 visiting ED	>4 months	262 patients required ED admission and presented with acute post-SARS-CoV-2 infection symptoms; 42.6% of ED visits resulted in hospitalization.<br>24% of ED visits (7% hospitalizations) occurred after 3 months from the initial COVID-19 diagnosis.
DeMartino 2022 [35]	• COVID-19<br>• Without COVID-19	6 months	Compared with controls, patients with COVID-19 were significantly more likely to have at least 1 inpatient admission through month 4 (all $p < 0.05$) and at least 1 ED visit through month 6 (all $p < 0.001$), though the magnitude of the difference between groups after month 1 was small (< 0.2% for inpatient, < 3% for ED).
Formoso 2023 [39]	• COVID-19<br>• Without COVID-19	Median 152 days	Previous exposure to the SARS-CoV-2 infection was associated with a higher probability of needing access to hospital care compared with patients without COVID-19 including non-surgical hospital admission (HR=1.38, 95%CI: 1.25-1.52), hospital admission for respiratory disease (HR=2.09, 95%CI: 2.07-4.09) and hospital admission for heart disease (HR=1.93, 95%CI: 1.46-2.55).
McNaughton 2022 [30]	• Positive SARS-CoV-2 tests results<br>• Negative SARS-CoV-2 test results	Mean 240 days	Patients with SARS-CoV-2 infection had 7.41 additional days hospitalized at the 99th percentile of the per-person-year rate compared with patients with negative SARS-CoV-2 test results. Rate ratios for the test-positive vs. test-negative comparison were statistically significant (rate ratio 1.50, 95%CI: 1.42-1.58).
Castriotta 2023 [36]	• COVID-19<br>• Without COVID-19	6 months	Exposed (vs. unexposed) individuals were more likely to be admitted to the hospital (overall pooled IRR=1.98, 95%CI 1.66-2.36), particularly those hospitalized and admitted to the ICU during acute infection, with small variations across epidemic phases.
Lo 2023 [26]	• COVID-19<br>• Without COVID-19	Up to 13 months	A higher percentage of patients who were hospitalized during their acute phase of COVID-19 needed readmission to the hospital during the follow-up period compared with matched controls without COVID-19 diagnosis. The difference between patients with COVID-19 and matched control was statistically significant up to 180 days after the index date.
Ayoubkhani 2021 [37]	• COVID-19<br>• General population	Mean 140 and 153 days for COVID-19 and controls, respectively	Of 47 780 individuals in hospital with COVID-19 over the study period, 29.4% were readmitted. These events occurred at rates of 766 (95%CI: 753-779) per 1000 person-years, which were 3.5 (3.4 to 3.6) times greater than those in matched controls.
Tisler 2022 [38]	• COVID-19 patients (hospitalized)<br>• Without COVID-19	12 months	Among previously hospitalized patients, the hospital readmission rate was 2.1 times greater in patients initially hospitalized for COVID-19, compared with patients without COVID-19, during a mean follow-up of 10 (rate per 1000 person-years was 315.4 and 148.3 for COVID-19 patients and control, respectively)
Gordon 2024 [58]	• COVID-19<br>• Without COVID-19	3-6 months after infection	In a US pediatric cohort study, inpatient hospitalization was slightly less common among the COVID-19 group (RD = 0.94, $p = 0.158$) in the 3–6-month period after the diagnosis.

Study	Study arm(s)	Follow-up	Results
**Outpatient and Emergency Services**			
**Long COVID**			
Hedberg 2022 [34]	• Long COVID;<br>• No long COVID	4-12 months after COVID-19 diagnosis	Monthly primary care visit rates after (vs. before) the SARS-CoV-2 acute infection were increased among three COVID-19 severity subgroups with a long COVID diagnosis compared with matched controls (nonhospitalized: 0.23, 95% CI 0.21–0.25, hospitalized: 0.14, 95% CI: 0.10–0.18, and ICU-treated patients: 0.10, 95% CI: 0.03–0.16).<br>For outpatient specialist care, the monthly visit rate was significantly increased among non-hospitalized individuals (0.09, 95% CI: 0.06–0.13 visits), but not for hospitalized (0.09, 95% CI: −0.01 to 0.19 visits) and ICU-treated individuals (0.18, 95% CI: −0.02 to 0.38 visits) in the difference-in-difference analysis.
Mu 2023 [29]	• Long COVID;<br>• Pre-pandemic (self-controlled);<br>• COVID only, no long COVID;<br>• Unaffected by pandemic (before Dec 21, 2019;<br>• Contemporary non-COVID	Mean from 395 to 780 days depending on study arm	Individuals with long COVID attended more outpatient appointments monthly (mean difference 0.28, SD=0.54 per person per month for long COVID) than all control groups, and placed second after COVID only, no long COVID for critical care and ED attendances (0.02, SD=0.56; and 0.06, SD=0.38 per person per month, respectively).<br>Annually, individuals with long COVID experienced more outpatient appointments (mean difference 3.32 SD = 6.50 per person per year for long COVID) than all control groups, and ranked second after COVID only, no LC for critical care and ED attendances (0.21, SD=6.76; and 0.77, SD=4.56 per person per year, respectively)
Kirchberger 2023 [49]	• Long COVID;<br>• No long COVID	Median 26 months	In the past year, most of the medical specialists, as well as physical therapists, psychologists/ psychotherapists, and non-medical practitioners, were visited significantly more often by persons with long COVID compared with individuals without long COVID.
Nehme 2022 [28]	• Post-COVID condition with chronification of symptoms;<br>• Post-COVID condition without chronification of symptoms;<br>• COVID-19 without post-COVID condition;<br>• No COVID-19	15 months	Symptom chronicity (lasting up to 15 months post-infection) led to increased healthcare service utilization, i.e. visits to the emergency room, visits to the primary care physician, and visits to specialists compared with individuals with post-COVID-19 without persistent symptoms, individuals without post-COVID-19, and those without positive SARS-CoV-2 test results.
Fung 2023 [24]	• Long COVID;<br>• Long flu	2 months	Outpatients with long COVID were more likely to have outpatient visits than patients with long Flu (mean 2.9 (SD 3.4) vs. 2.5 (SD 2.7) visits, adjusted IRR=1.09, 95%CI: 1.08-1.10, p<0.001).<br>Patients with long COVID had significantly fewer ED visits than long flu (P<0.001), probably because of reduced ED usage during the pandemic.
**Long-term HCRU in patients with COVID-19**			

Study	Study arm(s)	Follow-up	Results
Hernandez-Romieu 2021 [46]	• COVID-19 (non-hospitalized)	6 months	69% of patients had at least 1 outpatient visit during the 28–180 days after COVID-19 diagnosis (10% were due to an active COVID-19 diagnosis, and 3–7% were due to symptoms potentially related to COVID-19) and less than 2% required hospitalization.
DeMartino 2022 [35]	• COVID-19<br>• Without COVID-19	6 months	The proportion of individuals with outpatient visits decreased to a level similar to the control group in month 6. Among commercially insured patients, mental health and rehabilitation visits were relatively rare (< 7%), and the magnitude of the difference between COVID-19 and control groups was minimal. Among Medicare-insured patients, mental health and rehabilitation visits were also relatively rare; however, the COVID-19 group was significantly more likely to have mental health visits through month 5 (all p<0.01) and rehabilitation visits through month 4 (all p < 0.01) compared with controls. Patients with COVID-19 were significantly more likely to have at least 1 ED visit through month 6 (all p < 0.001), though the magnitude of the difference between groups after month 1 was small (< 3%).
Formoso 2023 [39]	• COVID-19<br>• Without COVID-19	Median 152 days	Patients with previous exposure to SARS-CoV-2 infection required increased outpatient specialist visits compared with those without COVID-19, particularly visits to pneumologists (HR=2.45, 95%CI: 2.17-2.76), cardiologists (HR=1.44, 95%CI: 1.33-1.55), neurologists (HR=1.25, 95%CI: 1.14-1.36), rheumatologists (HR=1.17, 95%CI: 1.05-1.31), and endocrinologists (HR=1.30, 95%CI: 1.21-1.39). Previous exposure to the SARS-CoV-2 infection was also associated with a higher probability of needing access to an emergency room compared with patients without COVID-19 (HR=1.36, 95%CI: 1.29-1.42).
Castriotta 2023 [36]	• COVID-19<br>• Without COVID-19	6 months	Patients hospitalized or admitted to ICU during their acute phase of COVID-19 had significantly higher rates of outpatient visits in the 6 months following infection compared with patients unexposed to COVID-19.
Lo 2023 [26]	• COVID-19<br>• Without COVID-19	Up to 13 months	A higher percentage of patients who were hospitalized during their acute phase of COVID-19 needed physician visits and ED or urgent care clinic visits compared with matched controls without a COVID-19 diagnosis during the follow-up period.
McNaughton 2022 [30]	• Positive SARS-CoV-2 tests results<br>• Negative SARS-CoV-2 test results	Mean 240 days	Patients with SARS-CoV-2 infection had statistically significant more outpatient visits (rate ratios 1.05, 95%CI: 1.04-1,05), ED visits (rate ratios 1,03, 95%CI: 1,01-1,05), and long-term care days (rate ratios 2,23, 95%CI: 2,01-2,48) compared with patients with negative SARS-CoV-2 test results.
Magnusson 2022 [63]	• Positive SARS-CoV-2 tests results<br>• Negative SARS-CoV-2 test results<br>• Untested controls	24 weeks	The percentage of pediatric patients diagnosed with COVID-19 who used primary care declined steadily from 1 week after positive SARS-CoV-2 test results; by 9–12 and 13–24 weeks, levels were similar to those observed before the SARS-CoV-2 infection. No increased use of specialist care was observed among the study groups.
Gordon 2024 [58]	• COVID-19<br>• Without COVID-19	3-6 months after infection	In a US pediatric cohort study, the mean rate (per 100 children) of outpatient office visits was slightly lower in the COVID-19 group (RD = 0.99; p = 0.008).

Study	Study arm(s)	Follow-up	Results
			ED utilization was similar between groups (RD=0.94; p=0.158)

Abbreviations: HR, hazard ratio; IRR, incidence rate ratio; ED, emergency department; ICU, intensive care unit; SD, standard deviation

Supplementary Table S6. Summary of study results on long-term healthcare costs in patients with long COVID or with persistent symptoms of COVID-19

Study	Study arm(s)	Follow-up	Results
**Overall**			
**Long COVID**			
Mu 2023 [29]	• Long COVID;<br>• Pre-pandemic (self-controlled);<br>• COVID only, no long COVID;<br>• Unaffected by pandemic (before Dec 21, 2019;<br>• Contemporary non-COVID	Mean from 395 to 780 days depending on study arm	The cost of care for patients with long COVID diagnoses during the follow-up was £3,335.40 per patient per year and was nearly four times higher than the cost of care for the same individuals before the pandemic and nearly three times more than the cost of care for matched patients without a COVID-19 diagnosis before and during the pandemic.
Tene 2023 [25]	• Long COVID;<br>• Non long COVID	12 months	The relative adjusted increase in mean total monthly healthcare costs among patients with long COVID compared to patients without long COVID diagnosis was 1.74, 1.72, and 1.89 at 4-, 6-, and 12-month follow-ups, respectively.
Patterson 2022 [55]	• Long COVID;<br>• Ongoing COVID symptoms (lasting 4 to 12 weeks after onset of the disease);<br>• Non long COVID (lasting <4 weeks after onset of the disease)	NA	Incremental all-cause and disease-specific costs for commercial long COVID patients compared with patients with duration of COVID-19 symptoms <4 weeks were $94,839 (95%CI: $88,029-$101,649) and $50,736 (95%CI: $45,337-$56,136), respectively.<br>Incremental all-cause and disease-specific costs for Medicare long COVID patients compared with patients with duration of COVID-19 symptoms <4 weeks were $48,036 (95%CI: $45,199-$50,872) and $20,124 (95%CI: $17,742-$22,507), respectively.
**Long-term costs in patients with COVID-19**			
Koumpias 2022 [32]	Self-controlled study: pre and post COVID-19 diagnosis	6 months before and after COVID-19 diagnosis	COVID-19 diagnosis was associated with an additional $223.59 in total monthly medical expenditures, corresponding to a 2.75-fold increase in total healthcare costs compared with the pre-diagnosis level. Total monthly medical expenditures declined gradually from month to month but remained higher than the pre-diagnosis costs after 6 months of follow-up.
Chambers 2023 [52]	• COVID-19;<br>• Without COVID-19	12 months	Among commercial members, from the baseline period to the post period, healthcare spending increased $41.61 (7.7%) per member per month more among COVID-19 cases compared with their matched controls.

Study	Study arm(s)	Follow-up	Results
			Among Medicare Advantage members, total healthcare spending increased $97.30 (13.1%) per member per month more among COVID-19 cases than their matched controls during the post-period compared with the baseline period.
Wolff Sagy 2023 [53]	• COVID-19;<br>• Without COVID-19	Median 9 months	The mean monthly cost incurred per COVID-19 recoverees over up to 15 months (median 9 months) of post-COVID-19 follow-up was higher by 8.2% (US$8.2) compared with matched controls. The excess cost attributable to post-COVID-19 effects was 7.6% of the cost in controls (US$7.7 per patient per month).
Pike 2023 [54]	• COVID-19;<br>• Without COVID-19	6 months	Among children, excess medical costs during 1, 3, and 6 months were $208 (95% CI: $136–$281), $549 (95% CI: $361–$736), and $1,011 (95% CI: $717–$1,306), respectively. Medical costs were, on average, 1.75 times higher among children with COVID-19 than among children without COVID-19 during the 3 periods. Among adults, excess medical costs during 1, 3, and 6 months were $393 (95% CI, $359–$427), $930 (95% CI, $857–$1,003), and $1,562 (95% CI, $1,451–1,673), respectively. Medical costs were, on average, 1.56 times higher among adults with COVID-19 than among adults without COVID-19 during the 3 periods.
DeMartino 2022 [35]	• COVID-19;<br>• Without COVID-19	6 months	Total healthcare costs in month 1 were significantly higher among patients with COVID-19 than controls (mean differences: $3,706 for commercial; $10,595 for Medicare; both $P < 0.001$), driven by inpatient costs.
Formoso 2023 [39]	• COVID-19;<br>• Without COVID-19	Median 152 days	Overall, the cost of care provided to those who had a previous SARS-CoV-2 infection was 27% higher (€10 357 221, mean: 287.41, range 0–114 610 vs 8 149 196, mean 226.14, range 0–69 143).
Khan 2023 [56]	• Hospitalized COVID-19<br>• Non-hospitalized COVID-19	4 months	Average total medical spending was 96%, 92%, and 68% higher for individuals who were hospitalized for COVID-19 vs. their non-hospitalized counterparts after 1 month, 2 months, and 4 months from the COVID-19 diagnosis date, respectively, excess costs were $11,242, $10,393, and $4,959 after 1, 2 and 4 months, respectively.
Gordon 2024 [58]	• COVID-19<br>Without COVID-19	3-6 months after infection	A slight but statistically significant decrease in total healthcare costs in the COVID-19 group compared with the control was observed.
**Inpatient Costs**			
**Long COVID**			
Walter 2022 [47]	• Long COVID	NA	In 2021, long COVID diagnosis was associated with total inpatient costs of €136,608,719, with a mean cost of €4,583 per case.
Tene 2023 [25]	• Long COVID;<br>• Non long COVID	12 months	Mean monthly costs of hospitalization during follow-up in patients with long COVID was $964.6 and $106.6 in patients without long COVID (HR=1.98 95%CI: 1.20-3.28)
**Long-term costs in patients with COVID-19**			

Study	Study arm(s)	Follow-up	Results
Koumpias 2022 [32]	• Self-controlled study: pre and post COVID-19 diagnosis	6 months before and after COVID-19 diagnosis	In the study increase in total healthcare costs during the post COVID-19 diagnosis period was driven primarily by financial costs associated with inpatient care ($95.29, 95% CI: $92.20-$98.39; p < 0.001).
Chambers 2023 [52]	• COVID-19;<br>• Without COVID-19	12 months	A 14.6% increase in inpatient healthcare spent was observed among US commercially insured COVID-19 patients compared with controls (excess costs of $5.39, difference-in-differences). Medicare Advantage patients experience a lesser increase in healthcare spending for inpatient services than control (-22.8%; decreased costs of -$16.13, difference-in-differences).
Wolff Sagy 2023 [53]	• COVID-19;<br>• Without COVID-19	Median 9 months	Inpatient (hospitalization days) costs were increased by 20.3% in COVID-19 patients compared with controls.
DeMartino 2022 [35]	• COVID-19;<br>• Without COVID-19	6 months	Mean inpatient costs rapidly declined after the first month but remained numerically higher in patients exposed to COVID-19 compared to controls across 6 months; the differences were significant for up to month 5 for commercially insured patients, and up to month 2 for Medicare patients.
**Outpatient and ED Costs**			
**Long COVID**			
Tene 2023 [25]	• Long COVID;<br>• Non long COVID	12 months	Mean monthly costs of emergency visits during follow-up in patients with long COVID was $40.6 and $8.1 in patients without long COVID (HR=1.25 95%CI: 1.11-1.41) and of outpatient visits $611 and $-79.2 (HR=1.98 95%CI: 1.41-2.78)
Tufts 2023 [57]	• Long COVID; Longer-term symptoms of COVID;<br>• Exposed to COVID-19;<br>• Unexposed to COVID-19	Min 12 weeks	Among patients with COVID-19, a long COVID diagnosis and reporting of longer-term symptoms were associated with an over 3- and 6-fold increase in primary care consultation costs compared with patients unexposed to COVID-19, with incremental costs increase of £30.50 and £57.60 per patient, respectively
**Long-term costs in patients with COVID-19**			
Ashman 2023 [40]	• Hospitalized COVID-19	6 month	Hospitalized COVID-19 patients incurred high outpatient costs. Outpatient care (i.e., visits with a registered COVID-19 diagnosis) during the 6 months of follow-up of 466 hospitalized COVID-19 patients amounted to €77,311.30 in Sweden.
Koumpias 2022 [32]	• Self-controlled study: pre and post COVID-19 diagnosis	6 months before and after COVID-19 diagnosis	During the post COVID-19 diagnosis period notable increases in medical expenditures for care delivered via telemedicine ($6.90, 95% CI: $6.72-$7.09; $P < 0.001$) and for cardiology services ($3.43, 95% CI: $3.18-$3.67; $P < 0.001$) was reported. An increase in emergency care costs was also observed of an additional $40.82 (95% CI: $39.01-$42.64; $P < 0.001$).
Chambers 2023 [52]	• COVID-19;<br>• Without COVID-19	12 months	A 14.8% increase in outpatient care spending and 11.4% in professional care spending were observed among US commercially insured COVID-19 patients compared with controls during the 12-month follow-up (excess costs were $22.65 and $21.26 for outpatient and professional services, respectively).

Study	Study arm(s)	Follow-up	Results
			Medicare Advantage patients experienced a greater increase in health care cost for outpatient service than controls (+44.9%) with an excess cost of $59.8.
Wolff Sagy 2023 [53]	• COVID-19;<br>• Without COVID-19	Median 9 months	Higher excess costs in patients with confirmed COVID-19 diagnosis compared to unexposed patients were observed for primary care (+7.5%), medical specialists' visits (+8.0%), paramedical professions visits (+8.0%), ED visits (+6.8%), and ambulatory care visits (+8.4%).
DeMartino 2022 [35]	• COVID-19;<br>• Without COVID-19	6 months	Outpatient and ED costs were higher in patients with COVID-19 compared with those without the infection for up to 6 months from diagnosis in the case of commercial insurance and up to 3-5 months (depending on the setting) in the case of Medicare.
**Pharmaceutical costs**			
**Long COVID**			
Tene 2023 [25]	• Long COVID;<br>• Non long COVID	12 months	Mean monthly costs of medication purchases during follow-up in patients with long COVID was $11.8 and $8.6 in patients without long COVID (HR=1.60 95%CI: 0.83-3.28).
**Long-term costs in patients with COVID-19**			
Chambers 2023 [52]	• COVID-19;<br>• Without COVID-19	12 months	A modest -6.4% (-$7.69) decrease in medication costs was observed among US commercially insured COVID-19 patients compared with controls. Medicare Advantage COVID-19 patients experienced a 19% ($27.73) increase in pharmacy costs compared with controls.
Wolff Sagy 2023 [53]	• COVID-19;<br>• Without COVID-19	Median 9 months	A modest -6.1% (-$0.53) decrease in medication costs was observed among COVID-19 patients compared with controls.
DeMartino 2022 [35]	• COVID-19;<br>• Without COVID-19	6 months	Pharmacy costs remained relatively constant in patients with COVID-19 diagnoses and were comparable to controls.
Gordon 2024 [58]	• COVID-19<br>• Without COVID-19	3-6 months after infection	A slight but statistically significant decrease in pharmacy costs in the COVID-19 group compared with the control was observed.

Abbreviations: HR, hazard ratio; ED, emergency department
